# Biochemical metabolic levels and vitamin D receptor *FokⅠ* gene polymorphisms in Uyghur children with urolithiasis

**DOI:** 10.1371/journal.pone.0212183

**Published:** 2019-02-11

**Authors:** Yuanni Huang, Qing Peng, Mian Bao, Caixia Liu, Kusheng Wu, Shuqin Zhou

**Affiliations:** 1 Department of Preventive Medicine, Shantou University Medical College, Shantou, Guangdong, China; 2 Department of Hepatobiliary Surgery II, Zhujiang Hospital of Southern Medical University, Guangzhou, Guangdong, China; 3 Department of Anesthesiology, the First People’s Hospital of Kashi, Kashi, Xinjiang, China; 4 Department of Anesthesiology, Zhujiang Hospital of Southern Medical University, Guangzhou, Guangdong, China; National Cancer Center, JAPAN

## Abstract

Because of lacking studies of urolithiasis in children, we detected the biochemical metabolic levels and *FokⅠ* polymorphisms in the vitamin D receptor (VDR) in Uyghur children with urolithiasis, and evaluated the associations of biochemical metabolic levels with *FokⅠ* genotypes. We included 142 Uyghur children (108 males) under age 14 years with a diagnosis of urolithiasis and 238 Uyghur children (154 males) under age 14 years without a history of urolithiasis as controls. Baseline information and data for serum and urine parameters were obtained from medical records. PCR-restriction fragment length polymorphism (PCR-RFLP) was used to analyze the VDR *FokⅠ* polymorphisms. In univariate analyses adjusting for age and sex, carbon dioxide combining power (CO_2_CP) (odds ratio [OR] = 1.13, 95% confidence interval [CI]: 1.07–1.19), serum magnesium (Mg) (OR = 1.27, 95% CI: 1.03–1.56) and serum chlorine (Cl) (OR = 0.93, 95% CI: 0.88–0.97) were related to Uyghur children urolithiasis risk. A multiple logistic regression model showed CO_2_CP (OR = 1.17, 95% CI: 1.09–1.26), levels of uric acid (OR = 1.01, 95% CI: 1.00–1.01) and serum sodium (Na) (OR = 0.90, 95% CI: 0.82–0.99) were associated with pediatric urolithiasis. The risk of urolithiasis was increased with the *F* versus *f* allele overall (OR = 1.42; 95% CI: 1.01–2.00) and for males (OR = 1.52, 95% CI: 1.02–2.27). However, metabolic levels did not differ by *FokⅠ* genotypes. In our population, CO_2_CP and levels of uric acid and serum Na as well as polymorphism of the *F* allele of the VDR *FokⅠ* may provide important clues to evaluate the risk of urolithiasis in Uyghur children.

## Introduction

Urolithiasis is a common urinary disease worldwide, with an enormous burden to the healthcare system. In recent decades, the incidence of urolithiasis, usually as an adult disease, has been increasing in children, with more prevalence in male than in female [1–4]. A recent review demonstrated that the annual incidence of urolithiasis in children has increased by approximately 6% to 10% during the last 25 years [5]. Simultaneously, the risk of urinary calculus recurrence is considerably high during childhood, with approximately 50% of children showing symptomatic recurrence at 3 years after a first stone [6]; such recurrence has a long-term adverse effect on kidney function. However, urolithiasis, whose morbidity varies widely in different regions of the world depending on race and geography [4], is often considered an endemic problem. Xinjiang, China, especially the southern part of Xinjiang, where the Uyghur population is mainly located, is a specific region with a high incidence of pediatric urolithiasis [7].

The factors contributing to the occurrence of urolithiasis among Uyghur children are complicated and mainly include socioeconomic status, racial difference, environmental condition, lifestyle change, diet habit, biochemical metabolic disturbance and genetic diversity [2,7–9]. Biochemical metabolic abnormalities play a crucial role in stone formation in children [10,11]. A high proportion of upper urinary tract stones was found correlated with biochemical metabolic disturbance, which may be derived from the dietary and environmental factors[12,13]. Hypercalciuria is one of the typical manifestations of biochemical metabolic abnormalities. Hypercalciuria may be the most important predisposing risk factor for calcium oxalate (CaOx) stones [14]. In addition, most stones were located in the upper urinary tract, with CaOx containing stones predominant in Uyghur children (even up to 91.1%) [7,15,16]. Thus, biochemical metabolism factors can provide a vital clue to determine calculus treatment and prevention [15].

In recent years, the genetic basis of urolithiasis has received increasing attention. Genome-wide association studies have revealed a possible association of the genes claudin 14 (*CLDN14*) and diacylglycerol kinase (*DGKH*) with urolithiasis in Caucasians (from Iceland and The Netherlands) and Japanese people, respectively [17,18]. Also, the vitamin D receptor (*VDR*) gene has been found related to risk of urolithiasis in some populations [19–21].

*VDR* gene, on 12q13.11, encodes a specific VDR that belongs to a nucleophilic protein, a critical player in calcium-phosphorus (Ca-P) metabolism and regulation of cell proliferation and differentiation [22,23]. The biological activity of vitamin D is mediated by binding to VDR in target cells. In response to hormone signal molecule binding, VDR regulates the transcriptional activity of 1, 25-dihydroxyvitamin D_3_ (1, 25(OH)_2_ D_3_)-responsive genes [23,24]. Several polymorphisms on *VDR* gene have been detected [19]. The thymine/cytosine (T/C) polymorphisms, one of the known *VDR* gene sites, are associated with two potential start codons (ATG/ACG), with the ATG producing a longer VDR sequence [22,24]. These T/C polymorphisms can be identified by restriction fragment length polymorphism (RFLP) with the enzyme FokⅠ, thereby resulting in two alleles, *F* and *f*, and three genotypes, *FF*, *Ff* and *ff* [22–24]. *FokⅠ* polymorphisms can change the VDR protein sequence to produce two proteins of different lengths; the sequence was recently reported as a candidate gene locus for some diseases, such as urolithiasis, prostate cancer and osteoporosis [19,24]. A new meta-analysis demonstrated that the *f* allele and *ff* genotype were related to urolithiasis risk in Asians [21]. Some researchers evaluated the association of *VDR* gene polymorphisms with metabolic disturbances in children with urinary stones [22,25].

The pathogenesis of urolithiasis is still unclear. Although research on adult urolithiasis was reported in Han populations in China and in Caucasians, the results were conflicting. Also, we lack knowledge of the biochemical metabolism and genetic factors in Uyghur children with urolithiasis in China. Here we determined biochemical metabolic levels and VDR *FokⅠ* polymorphisms in Uyghur children with urolithiasis in China, and evaluated the associations of biochemical metabolic levels with *FokⅠ* genotypes. We hoped to gain insights into the etiology and pathogenesis of children with urolithiasis and provide a theoretical basis for calculi treatment and prevention in this population.

## Materials and methods

### 2.1 Study participants

We included Uyghur outpatients and inpatients under age 14 years with a diagnosis of urolithiasis in the First People’s Hospital of Kashi in Xinjiang, China between April 2016 and February 2017. We also included Uyghur children (154 males, 84 females) without a history of stone formation from the same region as controls. Calculi were confirmed by ultrasonography, abdominal radiography or computerized tomography, and controls were confirmed to not have stones by abdominal radiography. Children with a diagnosis of chronic renal failure, urinary tract infection, urinary tract malformations, or chronic diarrhea or who were taking the drugs that affected metabolic level, such as calcium and vitamin D supplements, were excluded. Baseline characteristics (including demographic information, calculi locations and etc.) were obtained from medical records in the First People’s Hospital of Kashi. Likewise, the data for serum parameters such as uric acid, carbon dioxide combining power (CO_2_CP), urea, creatinine, Ca, P, magnesium (Mg), potassium (K), sodium (Na), chlorine (Cl); and urine factors such as pH were acquired from medical records. Serum CO_2_CP was detected by enzyme assay with a CO_2_CP assay kit (Beckman Coulter, USA), and the concentration of CO_2_CP was identified by use of an automatic biochemical analyzer (Beckman Coulter, USA). The written informed consents were obtained from all the parents or guardians of the children and partial children themselves who could understand the study after receiving detailed explanations of the study aim and potential consequences prior to enrollment. This study was performed with the approval of the Human Ethical Committee of Shantou University Medical College (Approval number: SUMC 2016XM-0017).

### 2.2 Analysis of VDR *FokⅠ* gene polymorphisms

DNA was isolated from peripheral blood collected in anticoagulant tubes with ethylene diaminetetraacetic acid by using a blood genomic DNA extraction kit (Tiangen Biotech Co., Beijing) according to the manufacturer’s protocol. The purity and concentration of extracted DNA were identified by using the NanoDrop 2000 Ultramicro Spectrophotometer (Thermo Scientific, USA), with the purity controlled in the range of 1.8–1.9.

Genotyping of the VDR *FokⅠ* was determined by conventional PCR-restriction fragment length polymorphism (PCR-RFLP) [19,22,24,26]. The primers were synthesized by Bgi Tech Co., with sequences from the literature as follows [26]: forward: 5’-CCTGGCACTGACTCTGGCTCTG-3’ and reverse: 5’-GGCTCCCTTCATGG AAACACC-3’. The PCR amplification was performed in 25 μL reaction mixtures that contained 12.5 μL 2×premix Taq (Takara Bio, Tokyo), 0.5 μL forward and reverse primers (10 μmol/μL mixtures), 1 μL DNA templates and 11 μL ddH_2_O with the following reaction program in a PCR thermal cycler (Bio-Rad, CA, USA): 98°C for 2 min, 35 cycles at 98°C for 10 sec, 59°C for 30 sec, 72°C for 1 min, and 72°C for 10 min.

**Fig 1:**
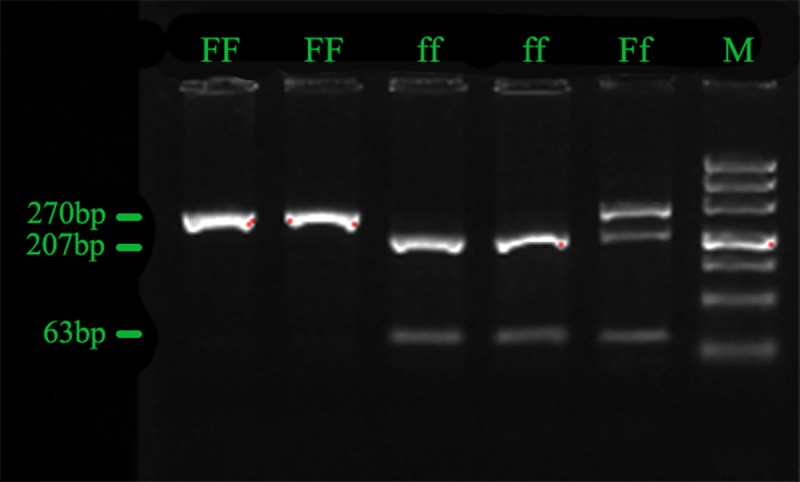
After digestion with the restriction enzyme FokⅠ,the DNA fragments were run on an agarose gel, and representative electrophoresis results of wild homozygote *FF* (270 bp), heterozygote *Ff* (270, 207, 63 bp) and mutant homozygote *ff* (207, 63 bp) are shown.

After the amplification, PCR products were digested by restriction endonuclease (FokⅠ) for 2 hr at 37°C, with 30-μL reaction mixtures containing 20 μL PCR products, 0.75 μL FokⅠ(New England Biolabs NEB-China, Beijing), 3 μL 10×NEBuffer and 6.25 μL ddH_2_O. The PCR products and digestion products were identified by electrophoresis on 2% and 3% agarose gel, respectively, and analyzed by using a gel imaging system (Bio-Rad, CA, USA). Genotypes of *FokⅠ* were visible on the basis of wild homozygote *FF* (270 bp), heterozygote *Ff* (270, 207, 63 bp) and mutant homozygote *ff* (207, 63 bp) [26] (Fig 1).

### 2.3 Statistical analysis

Continuous data are presented as mean ± SD and categorical data as frequency (%). Normality of the distribution of data was assessed by Kolmogorov-Smirnov and Shapiro-Wilk tests. Independent samples *t*-test and one-way ANOVA were used to compare continuous data when both normality and variance homogeneity were satisfied and otherwise, Mann-Whitney *U* and Kruskal-Wallis *H* tests. Pearson’s goodness-of-fit chi-square test was used to determine Hardy-Weinberg equilibrium in both patients and controls by using the online Encyclopedia for Genetic Epidemiology studies (OEGE) software (www.oege.org) [20]. The genotype and allele frequency distribution between two groups was evaluated by chi-square test. Unconditional multiple logistic regression was used to assess factors associated with urolithiasis, estimating odds ratios (OR) and 95% confidence intervals (CI). All statistical tests involved use of IBM SPSS 22.0 (IBM, Armonk, NY, USA), with two-sided *P* <0.05 considered statistically significant.

## Results

Among 142 children with calculi, up to 84.5% of stones were located in the upper urinary tract and only 15.5% in the lower urinary tract. The baseline characteristics and metabolic levels in children with stones and controls are in Table 1. The mean age of children with stones was 4.55±3.19 years (male/female ratio 3.18:1), and the mean age of the 238 controls was 5.02±3.50 years (male/female ratio 1.83:1). Levels of uric acid, creatinine and serum Ca, P, K and serum Na did not differ among 132 cases and 216 controls (all *P*>0.05), but urea and serum Cl levels were higher for children with stones than controls (*P* = 0.001 and 0.003, respectively) and CO_2_CP and serum Mg level were lower (*P*<0.001 and = 0.028). Urinary pH did not differ between children with stones and controls (*P*>0.05).

**Table 1:** Baseline characteristics and metabolic levels in children with stones and controls.

Characteristics	Children with stones (*n* = 142)	Control group (*n* = 238)	*P* value
Sex, *n* (%)			
Male	108 (76.1)	154 (64.7)	0.021
Female	34 (23.9)	84 (35.3)	
Age, years	4.55±3.19	5.02±3.50	0.257
Uric acid, umol/L	214.82±121.21	219.91±107.88	0.379
CO_2_CP, mmol/L	21.70±4.20	23.97±4.34	**<0.001**
Urea, mmol/L	5.42±3.68	4.35±1.71	**0.001**
Creatinine, umol/L	37.41±54.95	31.92±13.97	0.203
Serum Ca, mmol/L	2.32±0.13	2.29±0.19	0.081
Serum P, mmol/L	1.69±0.30	1.70±0.37	0.746
Serum Mg, mmol/L	0.85±0.10	0.88±0.14	**0.028**
Serum K, mmol/L	4.41±0.53	4.33±0.67	0.196
Serum Na, mmol/L	138.47±3.87	137.67±4.20	0.075
Serum Cl, mmol/L	105.21±4.29	103.62±5.19	**0.003**
Urinary pH	5.81±0.58	5.92±0.62	0.115

Data are described as mean with standard deviation unless indicated. CO_2_CP, carbon dioxide combining power; Ca: calcium; P: phosphate; Mg: magnesium; K: potassium; Na: sodium; Cl: chlorine; RBP, retinol binding protein

**Fig 2:**
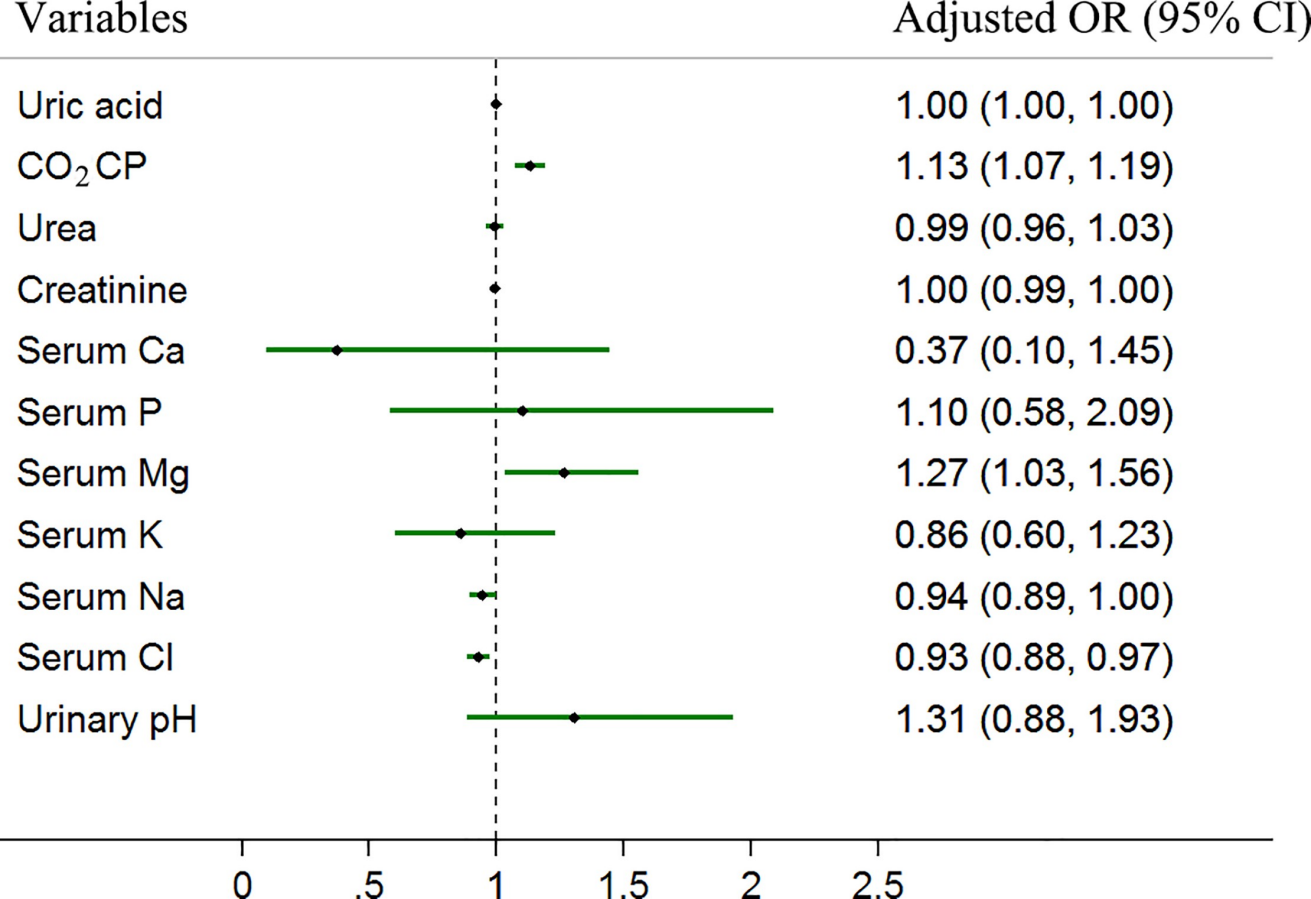
Metabolic factors associated with children urolithiasis risk after adjustment for age and sex.

After adjustment for age and sex, CO_2_CP (OR = 1.13, 95% CI: 1.07–1.19), serum Mg (OR = 1.27, 95% CI: 1.03–1.56) and Cl (OR = 0.93, 95% CI: 0.88–0.97) were associated with the development of children urolithiasis, while other factors (such as uric acid, urea, creatinine, serum Ca, P, K, Na and urinary pH) were not significantly different (Fig 2). Further multiple logistic regression model was performed including factors such as sex, age, uric acid, CO_2_CP, urea, creatinine, serum Ca, P, Mg, K, Na, Cl and urinary pH. The results showed that uric acid (OR = 1.01, 95% CI: 1.00–1.01), CO_2_CP (OR = 1.17, 95% CI: 1.09–1.26) and serum Na (OR = 0.90, 95% CI: 0.82–0.99) were significantly associated with children urolithiasis risk (Table 2).

**Table 2:** Multiple logistic regression analyses of factors associated with children urolithiasis risk.

Factors	*B*	Wald	*P*	OR (95%CI)
Sex				
Male				1.00 (Reference)
Female	-0.595	3.915	**0.048**	0.55 (0.31–0.99)
Age	0.045	1.048	0.306	1.05 (0.96–1.14)
Uric acid	0.005	5.936	**0.015**	1.01 (1.00–1.01)
CO_2_CP	0.158	17.453	**<0.001**	1.17 (1.09–1.26)
Urea	0.025	0.356	0.551	1.03 (0.95–1.11)
Creatinine	-0.017	3.598	0.058	0.98 (0.97–1.00)
Serum Ca	-1.164	1.380	0.240	0.31 (0.05–2.18)
Serum P	0.678	1.568	0.211	1.97 (0.68–5.69)
Serum Mg	0.195	1.950	0.163	1.22 (0.92–1.60)
Serum K	-0.163	0.387	0.534	0.85 (0.51–1.42)
Serum Na	-0.102	4.417	**0.036**	0.90 (0.82–0.99)
Serum Cl	0.013	0.111	0.739	1.01 (0.94–1.10)
Urinary pH	0.354	2.193	0.139	1.43 (0.89–2.28)

OR, odds ratio; 95% CI, 95% confidence interval

For *FokⅠ* polymorphisms, none of the genotype frequencies deviated from Hardy-Weinberg equilibrium in children with stones or controls (*χ*^2^ = 0 and 0.07, respectively). We could analyze the *FokⅠ* genotypes for 130 cases and 224 controls (Table 3). The *FF*, *Ff* and *ff* genotype frequencies were similar between children with stones and controls (*P* = 0.136). On unconditional logistic regression, the risk of urolithiasis was greater for children carrying *FF* and *Ff* genotypes than the *ff* genotype, although not significantly (OR = 2.11, 95% CI: 0.90–4.95 and OR = 1.36, 95% CI: 0.86–2.15, respectively). The *F* allele was more frequent in cases than controls (*P* = 0.045). The risk of urolithiasis was greater with the *F* than *f* allele (OR = 1.42; 95% CI: 1.01–2.00).

**Table 3:** Frequency distribution of the three genotypes (*FF*, *Ff* and *ff*) and two alleles (*F* and *f*) of the vitamin D receptor (VDR) *FokⅠ* gene in children with stones and controls.

	Children with stones (*n* = 130)	Control group (*n* = 224)	*P* value	OR (95%CI)
Genotype				
*FF*, *n (%)*	73 (56.2)	104 (46.4)	0.136	2.11 (0. 90–4.95)
*Ff*, *n (%)*	49 (37.7)	96 (42.9)		1.36 (0.86–2.15)
*ff*, *n (%)*	8 (6.1)	24 (10.7)		1.00 (Reference)
Allele				
*F*, *n (%)*	195 (75.0)	304 (67.9)	0.045	1.42 (1.01–2.00)
*f*, *n (%)*	65 (25.0)	144 (32.1)		1.00 (Reference)

Numbers do not add to the total because some participants were not successfully genotyped.

We analyzed the *FokⅠ* genotype and allele frequency distribution between children with stones and controls by sex (Table 4). The *F* allele was more frequent in cases than controls for males (*P* = 0.039). The risk of urolithiasis was greater for males with the *F* than *f* allele (OR = 1.52, 95% CI: 1.02–2.27, *P* = 0.039), but genotype frequency did not differ between males with stones and controls (*P* = 0.099). In contrast, genotype and allele frequency did not differ between females with stones and controls (*P* = 0.437 and 0.451, respectively).

**Table 4:** Frequency distribution of the three genotypes (*FF*, *Ff* and *ff*) and two alleles (*F* and *f*) of the VDR *FokⅠ* gene in children with stones and controls by sex.

	Male		*P* value	Female		*P* value
	Children with stones (*n* = 100)	Control group (*n* = 143)		Children with stones (*n* = 30)	Control group (*n* = 81)	
Genotypes						
*FF*, *n(%)*	55 (55.0)	65 (45.4)	0.099	18 (60.0)	39 (48.1)	0.437
*Ff*, *n(%)*	39 (39.0)	58 (40.6)		10 (33.3)	38 (46.9)	
*ff*, *n(%)*	6 (6.0)	20 (14.0)		2 (6.7)	4 (5.0)	
Alleles						
*F*, *n(%)*	149 (74.5)	188 (65.7)	0.039	46 (76.7)	116 (71.6)	0.451
*f*, *n(%)*	51 (25.5)	98 (34.3)		14 (23.3)	46 (28.4)	

We analyzed *FokⅠ* genotypes in children with stones and found no significant association of *FF*, *Ff* and *ff* genotypes with levels of uric acid, CO_2_CP, urea, creatinine; serum Ca, P, Mg, K, Na, Cl, or urinary pH (Table 5).

**Table 5:** Distribution of metabolic levels among the VDR *FokⅠ* genotypes in children with stones.

	*FF*, *n* = 65	*Ff*, *n* = 49	*ff*, *n* = 8	*P* value
Uric acid, umol/L	208.70±85.15	204.21±73.66	176.75±76.21	0.342
CO_2_CP, mmol/L	21.40±4.36	21.78±4.13	24.36±2.29	0.171
Urea, mmol/L	4.92±1.94	5.43±3.38	4.99±1.68	0.927
Creatinine, umol/L	33.21±27.55	33.82±25.58	29.60±4.95	0.848
Serum Ca, mmol/L	2.31±0.14	2.33±0.12	2.34±0.15	0.633
Serum P, mmol/L	1.67±0.27	1.68±0.32	1.62±0.38	0.993
Serum Mg, mmol/L	0.86±0.09	0.85±0.10	0.84±0.14	0.842
Serum K, mmol/L	4.41±0.52	4.35±0.54	4.62±0.41	0.365
Serum Na, mmol/L	138.43±4.01	138.09±3.61	139.87±4.19	0.480
Serum Cl, mmol/L	104.65±4.44	105.47±4.07	106.26±4.98	0.475
Urinary pH	5.87±0.69	5.78±0.42	5.50±0.38	0.285

Numbers do not add to the total because some patients were not successfully genotyped.

## Discussion

In this study, we measured biochemical metabolic levels and VDR *FokⅠ* polymorphisms in Uyghur children with urolithiasis in China, and evaluated the associations of biochemical metabolic levels with *FokⅠ* genotypes. CO_2_CP, levels of uric acid, and serum Na were associated with pediatric urolithiasis on a basis of multiple logistic regression analysis, and the *F* allele augmented the urolithiasis risk as compared with the *f* allele overall and in males.

To our knowledge, the results of previous studies concerning the association of VDR *FokⅠ* polymorphisms with urolithiasis in adults remained inconsistent and controversial due to differences in populations and races. For example, Liu et al. did not find any significant difference in allele or genotype frequencies for *FokⅠ* polymorphisms in a meta-analysis of the literature [23]. However, Zhou et al. found the *FokⅠ f* allele and *ff* genotype relevant in risk of urolithiasis for Asians in a meta-analysis of the literature [21]. Another meta-analysis demonstrated *FokⅠ* polymorphisms related to urolithiasis risk in Indian and Iranian people [27]. At present, few studies have been conducted to determine the association of the *FokⅠ* polymorphism with urolithiasis in children. In our specific study of Uyghur children with urolithiasis from Kashi, Xinjiang, we found the risk of urolithiasis increased 1.42-fold with the *F* than *f* allele (*P* = 0.045). Further analysis by sex showed that the *F* allele was a candidate factor for urolithiasis risk in males (*P* = 0.039).

As compared with Han Chinese and Caucasians, the Uyghur population is a special ethnic group. Archaeological findings indicated that the Uyghur have undergone migration from Europe to the Tarim Basin of Xinjiang, China, and assimilated and integrated with the Asian ethnic groups for hundreds of years [28]. Population genetic studies manifested the Uyghur population is a typical admixture of eastern Asian and European lineages, sharing mixed anthropological features and genetic traits of both Europeans and Asians [29–32]. A genetic study showed the Uyghur population was formed with 60% European ancestry and 40% Asian ancestry [30]. Thus, the *FokⅠ* genotype frequency distribution among Uyghur population may be unique.

Functionally, many biological functions of vitamin D in target cells of intestine, bone and kidney operate via VDR-mediated target-gene transcription regulation by hormone receptor complexes formed by 1,25(OH)_2_ D_3_ hormone signaling molecules with VDR. Also, the hormone receptor complexes affect the specific DNA sequences of target genes, regulating the expression of structure genes [33,34]. Thus, the variation in DNA sequence may affect the rate of gene transcription, stability of the mRNA and the activity of the receptor protein, finally resulting in diseases such as urolithiasis.

The *FokⅠ* polymorphisms of *VDR* gene in the potential start codons can change the nucleotide sequence, generating two VDR proteins of different lengths [24]. A full-length 427- amino acid VDR is designated either "*f*" to indicate the presence of the FokⅠrestriction site or "M1" for translation from the first methionine in the primary sequence; another shorter 424- amino acid VDR is denoted either "*F*" to indicate the absence of the FokⅠrestriction site or "M4" for translation from the methionine at the fourth position in the primary sequence [35,36]. Structurally, the *F* variant is three amino acids shorter compared to the *f* variant. Some functional studies were still inconclusive. A functional study found the *F* human VDR possesses more potent transcriptional activity [36]; another similar study also suggested the *F* variant (lacking the first three amino acids) interacted more efficiently with human basal transcription factor IIB (TFIIB) and had higher transcriptional activity than the *f* variant (full-length) [35]; but Gross et al. did not find any significant differences in ligand affinity, DNA binding, or transactivation activity in the two VDR forms expressed in COS-7 cells [37]. Surely, some epidemiological studies also suggested the *f* allele may carry a greater hazard regarding urolithiasis [21,38]. In our study, the result revealed an increased risk with the *F* allele for urolithiasis. The conflicting result indicates further functional studies in animals or cultured cells on urolithiasis.

Previously, with 24-hr urine analysis of metabolic parameters, most biochemical indicators (e.g., uric acid, Ca, Mg, P) were risk factors for urolithiasis in both adults and children [39,40], although this was debated in different studies. However, Ubetagoyena-Arrieta et al. determined some biochemical parameters (e.g., creatinine, urea, Na, K, Cl, uric acid, Ca, P, Mg and osmolality) in blood and 24-hr urine and suggested that the mean values of natriuresis, uricosuria, phosphaturia and magnesuria were significantly elevated in children with hypercalciuria versus controls, whereas the mean values of blood biochemical parameters were similar between cases and controls, with only the mean urea level significantly different [40]. In our work, serum CO_2_CP was strongly related to urolithiasis risk in both single-factor and multi-factor analyses, and uric acid and serum Na were associated with urolithiasis in a multiple regression analysis.

As literatures reported, a high percentage of upper urinary tract stones was associated with metabolic disturbance, which may stem from dietary patterns [13]. A epidemiological survey found the cow milk with higher Ca, P and other mineral contents was long-term fed to infants in the Kashi of Xinjiang, which may lead to an disorder of Ca and P metabolism, and then promoting the development of urolithiasis [41]. Also, more epidemiological investigations indicated high-protein, purine-rich food (such as animal offal, beef and mutton) and excessive intake of dietary sodium salt are common dietary patterns of Uyghur people [7,15,41]. A transient excessive consumption of high-protein and high-purine food could increase endogenous acid production and urinary excretion of calcium, oxalate, and uric acid, decreasing the excretion of citrate and increasing acid load in the body, and then decreasing urine pH and further resulting in the development of UA/ CaOx stones [7,13]. In addition, studies suggested CaOx stones can develop from UA-induced crystallization of calcium salts [42,43]. Thus, the data of serum CO_2_CP, uric acid may relate to the risk of urolithiasis. Previous study also found an increased intake of dietary sodium salt may increase the excretion of urinary calcium as well as CaOx-containing stone formation [15]. Surely, a higher sodium intake is accompanied by higher chlorine levels. Animal studies showed that a Cl^-^–oxalate exchanger, SLC26A6, could contribute to proximal tubule NaCl transport and mediate oxalate secretion in the intestine, thereby preventing CaOx stone formation [44]. More mechanism studies *in vitro* should be performed to confirm it.

Currently, studies of the association of serum biochemical metabolic levels with *FokⅠ* genotypes in children with urolithiasis are scarce. A similar study evaluated the association of urinary excretion levels with *FokⅠ* genotypes in children with urolithiasis and found no significant difference [22]. Likewise, we found no significant association of serum biochemical metabolic levels with *FokⅠ* genotypes. Mean biochemical levels differed among three genotype groups but not significantly.

Our study contains some limitations. First, theoretically, biochemical metabolic abnormalities may stem from environmental or dietary factors. Our study was retrospective and we could not investigate the diet of Uyghur children. Hence, we need to continue to track the source of metabolic abnormalities in future studies. Second, we did not collect 24-hr urinary samples of Uyghur children. Theoretically, 24-hr urinary samples could obtain a more reliable biochemical metabolic evaluation of urolithiasis such as urinary calcium than in blood samples. Finally, we did not collect laboratory values for serum 25-hydroxy and 1,25-dihydroxy vitamin D, which is possibly associated with the likely mechanism of CaOx stone occurrence. Future study is needed to determine the associations of these hormone levels with urolithiasis risk and *FokⅠ* mutations. Therefore, the calculus etiology lacks definite conclusions. Nevertheless, some biochemical metabolic parameters such as CO_2_CP, uric acid, serum Na level, and a *VDR* gene loci (*FokⅠ*) are associated with urolithiasis in children.

## Conclusion

The biochemical metabolic parameters such as CO_2_CP, uric acid and serum Na, and the *F* allele of the VDR *FokⅠ* may be candidate risk factors for urolithiasis risk in Uyghur children of China. Further understanding of the associations between these etiological factors and calculus components is needed to better prevent the occurrence and recurrence of pediatric urolithiasis.
